# Fibroblasts Impact Goblet Cell Responses to Lactic Acid Bacteria After Exposure to Inflammatory Cytokines and Mucus Disruptors

**DOI:** 10.1002/mnfr.201801427

**Published:** 2019-04-22

**Authors:** Chengcheng Ren, Jelleke Dokter‐Fokkens, Susana Figueroa Lozano, Qiuxiang Zhang, Bart J. de Haan, Hao Zhang, Marijke M. Faas, Paul de Vos

**Affiliations:** ^1^ Immunoendocrinology, Division of Medical Biology Department of Pathology and Medical Biology University of Groningen and University Medical Center Groningen 9700 RB Groningen The Netherlands; ^2^ School of Food Science and Technology Jiangnan University Wuxi 214122 China

**Keywords:** fibroblast, goblet cells, gut mucus barrier, lactic acid bacteria, modulatory properties

## Abstract

**Scope:**

Mucus produced by goblet cells contributes to gut barrier function. Lactic acid bacteria (LAB) have been shown to impact mucus production. It is not completely known whether mucus production is influenced by the abundantly present fibroblasts in the intestine.

**Methods and results:**

The influence of fibroblasts on mucus‐related genes including mucin‐2 (MUC2), trefoil factor 3 (TFF3), resistin‐like molecule β (RETNLB), carbohydrate sulfotransferase 5 (CHST5), and galactose‐3‐O‐sulfotransferase 2 (GAL3ST2) is examined after co‐culture of LS174T‐goblet cells and CCD‐18Co colonic fibroblasts in the presence and absence of LAB‐strains known to impact mucus function. This is also tested after exposure to TNF‐α, IL‐13, or the mucin synthesis inhibitor tunicamycin (Tm). Effects of fibroblasts are treatment duration‐ and bacterial species‐dependent under homeostatic conditions. During TNF‐α challenge, fibroblasts reverse *Lactobacillus (L.) rhamnosus* CCFM237‐elicited declined TFF3 expression. After IL‐13 exposure, *L. rhamnosus* CCFM237 and *L. fermentum* CCFM787 attenuate enhanced TFF3 and RETNLB expression, respectively, only in the presence of fibroblasts. LAB has no effects on Tm‐induced decreased expression of goblet cell‐related genes regardless of the presence of fibroblasts.

**Conclusion:**

It is demonstrated that goblet cell–fibroblast crosstalk impacts mucus synthesis and influences the effects of LAB on goblet cell‐related genes. Effects are LAB‐species and stressor dependent.

## 1 Introduction

The gut epithelial surface is covered by a protective mucus‐layer produced by intestinal goblet cells.^1^ This mucus layer forms an efficacious defensive barrier against the noxious luminal content.^2^ Mucin, as a key constituent of the mucus layer, endows mucus with its basic structural properties and forms a gel that contributes to the barrier function of the intestine.^2^ Mucin 2 (MUC2) is one of the most abundantly present gel‐forming mucins in the intestine.^2^ Mucin glycosylation and sulfation are necessary processes to ensure proper mucus barrier function.^3^, ^4^, ^5^ This was experimentally demonstrated in vivo where it was shown that mucin sulfation is a prerequisite for protection against colitis and helminth infection in mice.^3^, ^4^ During sulfation, the sulfate donor transfers sulfate to the sulfate acceptor. Goblet cells can express two sulfotransferases carbohydrate sulfotransferase 5 (CHST5) and galactose‐3‐O‐sulfotransferase 2 (GAL3ST2), which can catalyze the sulfate transport to the C‐6 position of *N*‐acetylgalactosamine and to the C‐3 position of galactose residues, respectively.^6^, ^7^

In addition to mucins, other goblet cell‐secreted molecules such as trefoil factor 3 (TFF3) and resistin‐like molecule β (RELMβ) are also crucial mucus components, contributing to the barrier functionality of mucus.^2^, ^5^ TFF3 is known to be of paramount importance for epithelial restitution and mucosal healing.^8^ It is reported that TFF3‐deficiency increased colitis susceptibility and impaired normal mucosal repair in dextran sulfate sodium‐administrated mice.^9^ RELMβ was confirmed to be implicated in dampening of intestinal inflammation and antagonizing nematode infection in the gut.^10^, ^11^

Barrier function of mucus can be impaired by luminal insults such as by pathogens, by food‐related mycotoxin deoxynivalenol, and by proinflammatory cytokines that impact the synthesis of mucus‐related components in goblet cells.^5^, ^6^, ^12^, ^13^ This has been shown to contribute to the pathogenesis of various intestinal disorders.^5^, ^6^ The T helper 1 type cytokine TNF‐α, which is a key mediator of gut inflammation in inflammatory bowel disease, adversely regulates mucus production and composition.^6^ IL‐13, a T helper 2 type cytokine with ability to promote mucin synthesis,^6^ mediates host resistance to gut nematode infection through modulating RELMβ production in goblet cells.^11^ Besides the immune mediators, microbial‐derived products such as tunicamycin (Tm) can perturb normal mucin glycosylation and induce ER stress in goblet cells, leading to abnormal goblet cell response and mucus synthesis.^14^

Gut commensal bacteria or specific food components may enhance barrier function by impacting goblet cell mucus synthesis. Intestinal microbial strain *Ruminococcus gnavus* E1 was shown to promote mucin expression and glycosylation.^15^ There is evidence from both in vivo and in vitro studies that specific probiotic lactic acid bacteria (LAB) strains beneficially regulate mucus production in goblet cells.^16^, ^17^ A specific LAB strain, i.e., *Lactobacillus (L.) plantarum* 299v, elevated mucin expression, which might have contributed to inhibition of pathogen adherence to intestinal epithelial cells.^16^ In one of our in vivo studies, this supportive effect of LAB was shown to be very species‐dependent as *L. plantarum* WCFS1 prevented age‐induced mucus barrier dysfunction in fast aging mice, but two other bacterial species *Bifidobacterium breve* and *L. casei* did not effectively preserve mucus function in the aged mice with intestinal inflammation as a consequence.^17^ These observations suggest that it is mandatory to screen LAB strains before application in mucus supportive foods.

Goblet cells react on luminal insults or on beneficial food components in close crosstalk with several cells in the intestine. An essential cell type that has gained not more than minor attention in crosstalk between goblet cell and other cell types in the gut is the abundantly present intestinal fibroblasts. Intestinal fibroblasts are a subset of stromal cells in the lamina propria located adjacent to gut epithelium.^18^ Fibroblast–gut epithelium crosstalk has been studied in the intestine and is of crucial importance for maintaining gut immune equilibrium.^18^ Fibroblasts interact with gut epithelium through production of growth factors such as fibroblast growth factor 7 (FGF7), hepatocyte growth factor (HGF), and insulin‐like growth factor 2 (IGF2), which are reported to regulate epithelial proliferation and differentiation but also impact mucus production.^19^, ^20^, ^21^, ^22^, ^23^ However, crosstalk of fibroblasts with goblet cells still remains largely unexplored. It is, for example, unknown whether intestinal fibroblasts modulate mucus synthesis by goblet cells and whether fibroblasts interfere with the previously reported LAB‐induced regulation of mucus genes in goblet cells.^24^

Therefore, the current study was designed to determine possible effects of fibroblasts on the expression of mucus function‐related genes (MUC2, TFF3, RETNLB, CHST5, and GAL3ST2) in goblet cells via a co‐culture model of the LS174T intestinal goblet cell line and CCD‐18Co colonic fibroblasts. Furthermore, we determined whether fibroblasts influenced the modulatory effects of different LAB strains on gene expression in goblet cells with or without exposure to TNF‐α, IL‐13, or a mucin synthesis disruptor Tm. In addition, in order to gain more mechanistic insights in the effects of interactions between goblet cells and fibroblasts on mucus regulation, alterations of the growth factor genes (FGF7, HGF, and IGF2) in CCD‐18Co fibroblasts were studied during their co‐culture with LS174T goblet cells.

## 2 Experimental Section

### 2.1 Cell Culture

Human colorectal adenocarcinoma cell line LS174T with a goblet cell‐like phenotype^25^ and CCD‐18Co human colonic fibroblasts were obtained from American Type Culture Collection (ATCC), and maintained in MEM eagle (EMEM) medium (Lonza, Verviers, belgium) supplemented with 10% fetal bovine serum (Sigma–Aldrich, St. Louis, MO USA), 2 mm l‐glutamine (Lonza, Verviers, Belgium), 60 µg mL^–1^ gentamicin sulfate (Lonza, Verviers, Belgium). Cells were cultured in humidified 5% CO_2_ atmosphere at 37 °C according to the protocol supplied by the manufacturer.

### 2.2 Bacterial Culture and Preparation

Bacterial strains applied in the present study were supplied by Culture Collections of Food Microbiology (CCFM), and described in **Table** 1. All strains were cultured statically at 37 °C in De Man–Rogosa–Sharpe (MRS) broth (Merck, Darmstadt, Germany) until reaching stationary phase. Bacterial suspension stocks used for stimulation experiments were prepared as previously described.^26^

**Table 1:** Bacterial strains applied in this study

Species	Strain designation	Reference or origin
*Lactobacillus plantarum*	CCFM^a^ 634	CGMCC^b^ 9740; Chinese Sichuan pickle
*Lactobacillus fermentum*	CCFM787	not available
*Lactobacillus reuteri*	CCFM14	CICC^c^ 6226; Yoghurt starter strain
*Lactobacillus acidophilus*	CCFM137	human feces
*Streptococcus thermophilus*	CCFM218	Kefir
*Lactobacillus rhamnosus*	CCFM237	CGMCC7317

a CCFM, Culture Collections of Food Microbiology, Jiangnan University, Wuxi, China

b CGMCC, China General Microbiological Culture Collection Center, Beijing, China

c CICC, China Center of Industrial Culture Collection, Beijing, China.

### 2.3 Monoculture and Co‐Culture Cell Models

In both monoculture and co‐culture cell models, 0.5 mL of LS174T cell suspension (3.5 × 10^5^ cells mL^–1^) was seeded on 12 mm diameter polyester transwell inserts with 0.4 µm pore size (Corning, Kennebunk, ME, USA), and cultured until reaching 70–80% confluence in the absence of fibroblasts. CCD‐18Co fibroblasts were seeded at 8 × 10^4^ cells per well in 12‐well plates (Corning, New York, USA), and cultured alone until forming confluent monolayers. Afterward, when starting stimulation experiments for the co‐culture model transwell inserts with LS174T cells were transferred in 12‐well plates with a confluent fibroblast monolayer. Culture medium in the wells was then renewed with 1.5 mL of fresh medium. In the LS174T‐seeded transwell inserts, medium was replaced by 0.5 mL of fresh medium with or without different stimuli. For bacterial stimulation, initial bacterial suspension stocks were diluted in cell culture medium at a final concentration of 2.3 × 10^7^ CFU mL^–1^. For cytokines and Tm challenges, cell culture medium was supplemented with 50 ng mL^–1^ recombinant human TNF‐α (ImmunoTools, Friesoythe, Germany), 25 ng mL^–1^ recombinant human IL‐13 (ImmunoTools, Friesoythe, Germany) or with 1 µg mL^–1^ Tm (Sigma–Aldrich). Fresh culture medium supplemented with different stimuli was applied in the apical compartment with LS174T cells. Cells were then incubated with different stimuli for the time periods as described in the figure legends.

### 2.4 RNA Extraction and Quantitative Reverse‐Transcription PCR

LS174T and CCD‐18Co cells were homogenized separately using TRIzol reagent (Life Technologies, Carlsbad, USA) at the end of stimulation. Total RNA extraction and cDNA synthesis using SuperScript II Reverse Transcriptase Kit (Invitrogen, Carlsbad, USA) were performed following the manufacturer's instructions. Quantitative PCR (qPCR) was carried out with qPCR Mastermix Plus (Eurogentec, Seraing, Belgium) and TaqMan Gene Expression Assays (Applied Biosystems, Foster City, USA) using ViiA7 Real‐Time PCR System (Applied Biosystems) as previously described.^24^ For LS174T cells, genes MUC2, TFF3, RETNLB, CHST5, GAL3ST2, and GUSB were quantified with TaqMan Gene Expression Assays as previously reported.^25^ For CCD‐18Co cells, TaqMan Gene Expression Assays were applied for genes FGF7 (KGF, Hs00940253_m1), HGF (Hs00300159_m1), IGF2 (Hs01005963_m1), and GAPDH (Hs99999905_m1). Expression levels of target genes in LS174T and CCD‐18Co cells were normalized to housekeeping gene GUSB^25^ and GAPDH,^27^ respectively. The 2^−△△Ct^ method was used for calculating fold change in gene expression levels versus untreated or vehicle controls.

### 2.5 Statistical Analysis

All results are shown as mean ± SD. Statistical analysis were performed by using GraphPad Prism version 6.0 (San Diego, CA, USA). Parametric distribution of data was tested using Shapiro–Wilk normality test. Statistical significance was determined by paired *t*‐test and one‐way analysis of variance (ANOVA) with Bonferroni multiple comparisons test for post hoc comparison where appropriate. Values of *p *< 0.05 were considered as statistically significant while *p *< 0.1 was considered a trend. ^#,^*p < 0.05; ^##,^**p < 0.01; ^###,^***p < 0.001.

## 3 Results

### 3.1 Fibroblasts Impacted Production of Mucus Function‐Related Genes in Goblet Cells in a Time‐Dependent Way

**Figure 1:**
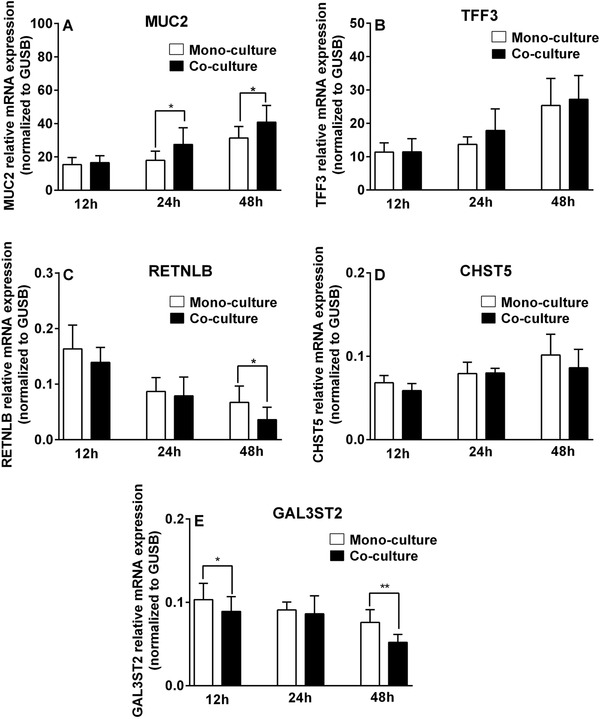
Expression of mucus‐function‐associated genes in goblet cells was altered in the presence of fibroblasts. MUC2, TFF3, RETNLB, CHST5, and GAL3ST2 mRNA expression levels in LS174T goblet cells cultured alone (monoculture) or with CCD‐18Co fibroblasts (co‐culture) for 12, 24, and 48 h were determined by real‐time RT‐PCR. Relative basal mRNA levels normalized to the housekeeping gene GUSB are expressed as 2^−△Ct^. Results are presented as mean and SD of five independent experiments. Paired *t*‐test was used to analyze statistical significance between monoculture group and co‐culture group at individual time points (*p < 0.05; **p < 0.01).

Intestinal fibroblasts, an essential type of stromal cell for gut homeostasis, have been proposed to support epithelial functions,^18^ but it is unknown whether these cells influence mucus production by goblet cells. To determine whether fibroblasts influence mucus production, we investigated the expression of goblet cell function‐associated genes (MUC2, TFF3, RETNLB, CHST5, and GAL3ST2) in the LS174T goblet cell line in the presence and absence of CCD‐18Co intestinal fibroblasts. Gene expression was studied after 12, 24, and 48 h of co‐culturing. As shown in **Figure** 1, fibroblasts impacted specific goblet cell‐associated gene expression in a time‐dependent fashion. The presence of fibroblasts significantly enhanced MUC2 expression by 52.5% and 30.7%, respectively, after 24 and 48 h of co‐culturing (*p *< 0.05 for both 24 and 48 h; Figure 1A). In addition to MUC2, TFF3 mRNA level was up‐regulated 30.5% in LS174T goblet‐cells following 24 h co‐culture with CCD‐18Co fibroblasts but this did not reach statistical significance (Figure 1B). In contrast, fibroblasts induced a significant decrease in RETNLB expression at 48 h (*p *< 0.05; Figure 1C). Moreover, GAL3ST2 transcription in LS174T goblet cells was significantly reduced by 12 h (*p *< 0.05) and 48 h (*p *< 0.01) of co‐culture with CCD‐18Co fibroblasts (Figure 1E). Co‐culture of LS174T cells with CCD‐18Co fibroblasts did not alter CHST5 expression (Figure 1D).

### 3.2 LAB Conferred Differential Regulation of Mucus Function‐Related Genes in Goblet Cells Co‐Cultured with Fibroblasts in a Species‐ and Treatment Duration‐Dependent Fashion

**Figure 2:**
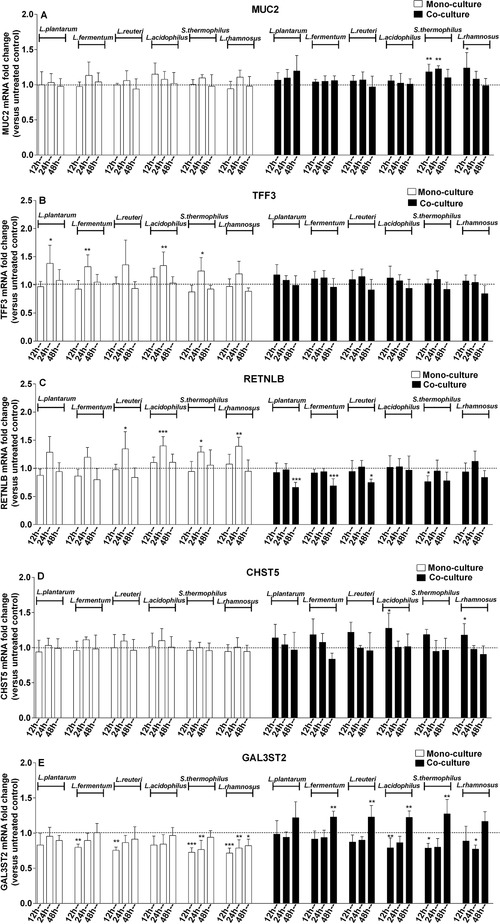
LAB differentially modulated goblet cell‐related gene expression in the presence of intestinal fibroblasts in a species‐ and time‐dependent manner. MUC2, TFF3, RETNLB, CHST5, and GAL3ST2 expression levels in LAB (2.3 × 10^7^ CFU mL^–1^)‐stimulated LS174T goblet‐cells cultured with (co‐culture) or without CCD‐18Co fibroblasts (monoculture) were determined by real‐time RT‐PCR following 12, 24, and 48 h of bacterial treatment. Gene expression levels were normalized to the housekeeping gene GUSB and presented as fold change relative to respective unstimulated control groups (monoculture or co‐culture control). Results are reported as mean and SD of five independent experiments. One‐way ANOVA with Bonferroni multiple comparisons test was used for testing statistical significance between bacterial treatment groups and untreated control groups (*p < 0.05; **p < 0.01, ***p < 0.001).

We previously showed that LAB modulate mucus‐related genes in goblet cells.^24^ Here, we further investigated whether fibroblasts affect the regulatory properties of LAB on these mucus‐related genes. To this end, transcription levels of mucus‐related genes in LAB‐stimulated LS174T goblet‐cells cultured with or without CCD‐18Co fibroblasts were studied following 12, 24, and 48 h of LAB treatment. As shown in **Figure** 2, there was a clear effect of LAB on mucus‐related genes, which was different in the presence of fibroblasts. In the absence of fibroblasts, following 24 h of stimulation, LAB strains significantly enhanced TFF3 (*p *< 0.05 for *L. plantarum* CCFM634 and *Streptococcus (S.) thermophilus* CCFM218; *p *< 0.01 for *L. fermentum* CCFM787 and *L. acidophilus* CCFM137) and RETNLB transcription (*p *< 0.05 for *L. reuteri* CCFM14 and *S. thermophilus* CCFM218; *p *< 0.01 for *L. rhamnosus* CCFM237; *p *< 0.001 for *L. acidophilus* CCFM137). The presence of fibroblasts reversed this effect of these LAB strains (Figure 2B,C). In the presence of fibroblasts, expression levels of MUC2 (*p *< 0.05 for *L. rhamnosus* CCFM237 at 12 h; *p *< 0.01 for *S. thermophilus* CCFM218 at 12 and 24 h), CHST5 (*p *< 0.05 for *L. acidophilus* CCFM137 and *L. rhamnosus* CCFM237; both at 12 h), and GAL3ST2 (*p *< 0.01 for *L. fermentum* CCFM787, *L. reuteri* CCFM14, *L. acidophilus* CCFM137, and *S. thermophilus* CCFM218; all at 48 h) were significantly elevated by specific LAB strains, which did not occur when LS174T goblet‐cells were cultured alone (Figure 2A, D, and E).

Furthermore, modulatory properties of LAB on gene expression were species‐dependent in LS174T goblet‐cells with or without culturing with CCD‐18Co fibroblasts. We found that MUC2 expression was only markedly augmented by two out of six tested species, i.e., by *S. thermophilus* (*p *< 0.01 at 12 and 24 h) and *L. rhamnosus* (*p *< 0.05 at 12 h) when fibroblasts were present (Figure 2A). For TFF3, after 24 h of LAB treatment only specific species including *L. plantarum* (*p *< 0.05), *L. fermentum* (*p *< 0.01), *L. acidophilus* (*p *< 0.01), and *S. thermophilus* (*p *< 0.05) elicited a significant increase in its expression when LS174T goblet‐cells were cultured without fibroblasts (Figure 2B). Notably, the expression of goblet cell‐related genes was time‐dependently modulated by LAB in LS174T goblet cells cultured with or without CCD‐18Co fibroblasts. For instance, under LS174T goblet‐cell monoculture conditions remarkably heightened RETNLB transcription was only obtained with 24 h of LAB treatment (*p *< 0.05 for *L. reuteri* CCFM14 and *S. thermophilus* CCFM218; *p *< 0.01 for *L. rhamnosus* CCFM237; *p *< 0.001 for *L. acidophilus* CCFM137; Figure 2C). Moreover, under LS174T/CCD‐18Co co‐culture conditions specific LAB strains such as *L. acidophilus* CCFM137 (*p *< 0.05) and *L. rhamnosus* CCFM237 (*p *< 0.05) significantly potentiated CHST5 expression levels only after 12 h of LAB stimulation (Figure 2D).

### 3.3 Fibroblast Growth Factor Genes were Modulated by Co‐Culture with (LAB‐stimulated) Goblet Cells

**Figure 3:**
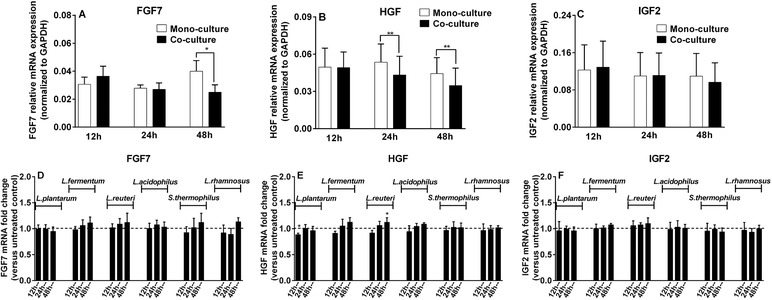
Expression of growth factor genes in intestinal fibroblasts was regulated when in co‐culture with (LAB‐stimulated) goblet cells. A, B, C) Basal expression levels of FGF7, HGF, and IGF2 in CCD‐18Co fibroblasts cultured with (co‐culture) or without LS174T goblet cells (monoculture) for 12, 24, and 48 h. Gene expression levels were normalized to the housekeeping gene GAPDH and are presented as 2^−△Ct^. D, E, F). Fold change in mRNA levels of fibroblast growth factor genes induced by LAB (2.3 × 10^7^ CFU mL^–1^)‐stimulated LS174T goblet‐cells over co‐culture untreated control group was calculated using 2^−△△Ct^ method. All results are presented as mean and SD of five independent experiments. For basal expression data (A, B, and C), paired *t*‐test was used to test statistical significance between monoculture group and co‐culture group at individual time points. For fold change data (D, E, and F), significance between bacterial stimulation groups and respective untreated control group was determined using one‐way ANOVA with Bonferroni multiple comparisons test (*p < 0.05; **p < 0.01).

Intestinal fibroblasts‐derived growth factors such as FGF7, HGF, and IGF2 are suggested to play a role in modulating epithelial cell functions such as enhancing cell proliferation but also effects on mucus production have been demonstrated.^19^, ^20^, ^21^, ^22^, ^23^ Therefore, we investigated whether fibroblast‐induced modulation of mucus‐related genes can be attributed to altered expression of fibroblast growth factors during fibroblast/goblet cell co‐cultures. To address this, basal mRNA levels of growth factor genes (FGF7, HGF, and IGF2) in CCD‐18Co fibroblasts cultured with or without LS174T goblet‐cells were first measured to study whether and how goblet cells modulated genes transcription in fibroblasts. We observed that FGF7 expression was significantly suppressed in CCD‐18Co fibroblasts after 48 h co‐culture with LS174T goblet cells, as compared to expression levels in the absence of LS174T goblet cells (*p *< 0.05; **Figure** 3A). In addition to FGF7, goblet cells also induced a significant decrease in HGF expression at 24 and 48 h (*p *< 0.01 for both 24 and 48 h; Figure 3B).

Next, to determine whether LAB exposure influences the expression of fibroblast genes, we quantified relative transcription levels of FGF7, HGF, and IGF2 in CCD‐18Co fibroblasts cultured with LAB‐treated LS174T goblet cells, as compared to expression levels in LS174T/CCD‐18Co co‐culture group without LAB treatment (unstimulated co‐culture control group). As shown in Figure 3, LAB stimulation only regulated the expression of HGF, and the effects of LAB were species‐specific and treatment period‐dependent. For instance, *L. plantarum* CCFM634 decreased HGF expression only at 12 h of stimulation (*p *< 0.05; Figure 3E). However, significantly enhanced HGF transcription only appeared following 48 h incubation with *L. reuteri* CCFM14‐stimulated LS174T goblet cells (*p *< 0.05; Figure 3E).

### 3.4 Goblet Cell‐Associated Genes During TNF‐α Treatment were Differentially Modulated by LAB in the Absence and Presence of Fibroblasts

**Figure 4:**
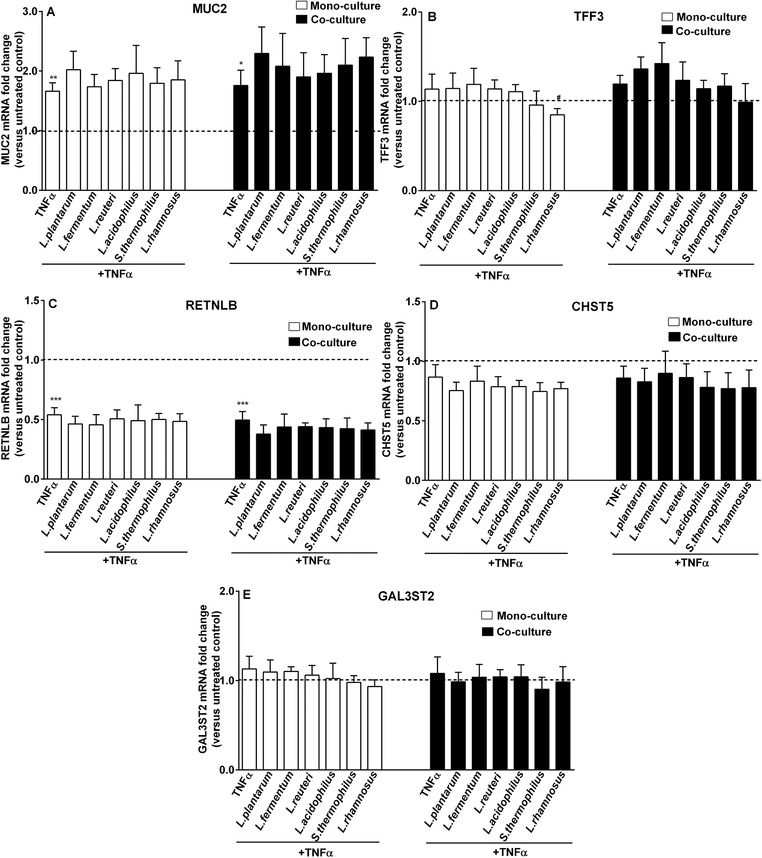
Goblet‐cell‐associated genes during TNF‐α exposure were divergently modulated by LAB in the presence and absence of fibroblasts. LS174T goblet‐cells were simultaneously stimulated with TNF‐α (50 ng mL^–1^) and various bacterial strains (2.3 × 10^7^ CFU mL^–1^) for 24 h with (co‐culture) or without co‐culture with CCD‐18Co fibroblasts (monoculture). Expression of mucus function‐related genes (MUC2, TFF3, RETNLB, CHST5, and GAL3ST2) in LS174T goblet cells was assessed and presented as fold change relative to respective untreated control groups (monoculture or co‐culture untreated control). Results are shown as mean and SD of five independent experiments. One‐way ANOVA with Bonferroni multiple comparisons test was used for statistical comparisons (*vs untreated control; ^#^vs TNF‐α group; ^#,^*p < 0.05; **p < 0.01; ***p < 0.001).

**Figure 5:**
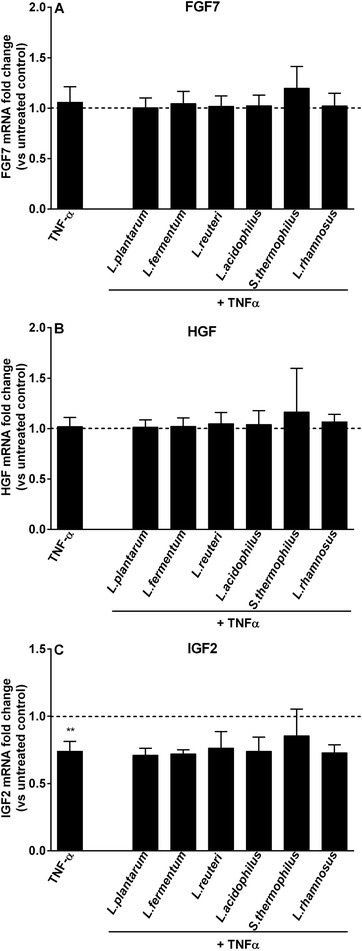
LAB‐mediated modulation of growth factor genes transcription in fibroblasts co‐cultured with goblet cells during TNF‐α challenge. CCD‐18Co fibroblasts were cultured with LS174T goblet cells (co‐culture), which were concomitantly stimulated with LAB (2.3 × 10^7^ CFU mL^–1^) and TNF‐α (50 ng mL^–1^) for 24 h. FGF7, HGF, and IGF2 expression levels in CCD‐18Co fibroblasts were assessed at the end of stimulation. Results are calculated as fold change in mRNA levels of growth factor genes against co‐culture untreated control group, and represent mean and SD of five independent experiments. One‐way ANOVA with Bonferroni multiple comparisons test was used to determine statistical significance (*vs co‐culture untreated control; **p < 0.01).

Proinflammatory cytokines have been suggested to impact goblet cell‐related genes, and this may induce intestinal barrier disruption.^6^ As fibroblasts may impact cytokine‐conferred modulatory effects on goblet cell‐related genes, here we first investigated the effect of T helper 1 cytokine TNF‐α in the presence of CCD‐18Co fibroblasts. In addition, to study whether modified expression of fibroblast growth factor genes is responsible for the expression difference in goblet cell‐related genes during TNF‐α stimulation in the presence and absence of fibroblasts, transcription levels of fibroblast growth factor genes (FGF7, HGF, and IGF2) were analyzed in TNF‐α‐treated LS174T/CCD‐18Co co‐culture models. We found that MUC2 expression was upregulated by TNF‐α under LS174T monoculture condition (*p *< 0.01 vs monoculture untreated control; **Figure** 4A), and TNF‐α‐induced elevated MUC2 expression levels were further increased by fibroblasts (*p *< 0.01; monoculture TNF‐α versus co‐culture TNF‐α; Figure S1, Supporting Information). In contrast to the increasing effects on MUC2 expression, TNF‐α stimulation remarkably inhibited RETNLB transcription in both LS174T monoculture and LS174T/CCD‐18Co co‐culture systems (*p *< 0.001 vs respective untreated controls; Figure 4C). Fibroblasts induced a trend toward further downregulating RETNLB expression during TNF‐α stimulation (*p* = 0.054, monoculture TNF‐α vs co‐culture TNF‐α; Figure S1, Supporting Information). For gene expression in fibroblasts, we observed that in LS174T/CCD‐18Co co‐cultures only IGF2 expression was lowered in response to TNF‐α stimulation (*p *< 0.01 vs co‐culture untreated control; **Figure** 5C).

Since we previously showed that LAB could partly prevent TNF‐α‐induced modifications in goblet cell‐related genes expression,^24^ we also studied the contribution of fibroblasts on LAB‐induced effects on goblet cell‐related genes during TNF‐α stimulation. In the absence of fibroblasts, *L. rhamnosus* CCFM237 treatment significantly reduced TFF3 expression during TNF‐α challenge (*p *< 0.05 vs monoculture TNF‐α), and this effect disappeared in the presence of fibroblasts (Figure 4B). Besides, expression of growth factor genes (FGF7, HGF, and IGF2) was also assessed in fibroblasts when co‐culture with LAB‐stimulated LS174T cells. It was found that LAB addition did not influence the expression of fibroblast genes in TNF‐α‐challenged LS174T/CCD‐18Co co‐culture system (Figure 5).

### 3.5 LAB Differently Regulated Goblet Cell‐Associated Genes During IL‐13 Treatment in the Absence and Presence of Fibroblasts

**Figure 6:**
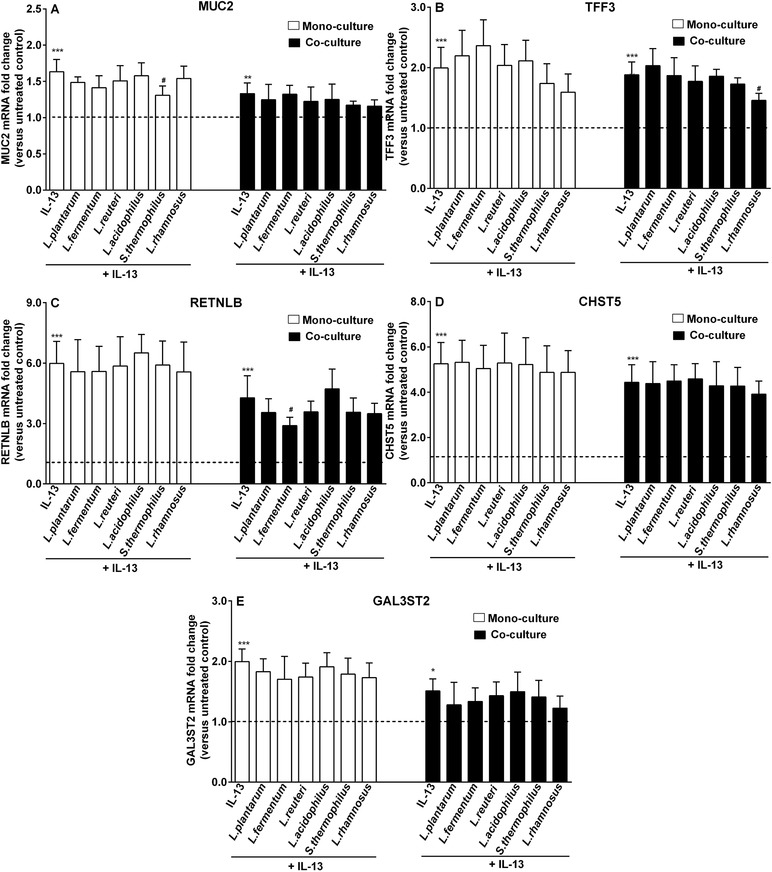
Goblet‐cell‐related genes during IL‐13 exposure were differentially regulated by LAB in the presence and absence of fibroblasts. LS174T goblet cells were concurrently stimulated with IL‐13 (25 ng mL^–1^) and different bacterial strains (2.3 × 10^7^ CFU mL^–1^) for 48 h with (co‐culture) or without the presence of CCD‐18Co fibroblasts (monoculture). Goblet‐cell‐associated genes (MUC2, TFF3, RETNLB, CHST5, and GAL3ST2) expression was measured and reported as fold change compared to respective untreated control groups (monoculture or co‐culture untreated control). Results are presented as mean and SD of five independent experiments. One‐way ANOVA with Bonferroni multiple comparisons test was used for statistical comparisons (*vs untreated control; ^#^vs IL‐13 group; ^#,^*p < 0.05; **p < 0.01; ***p < 0.001).

**Figure 7:**
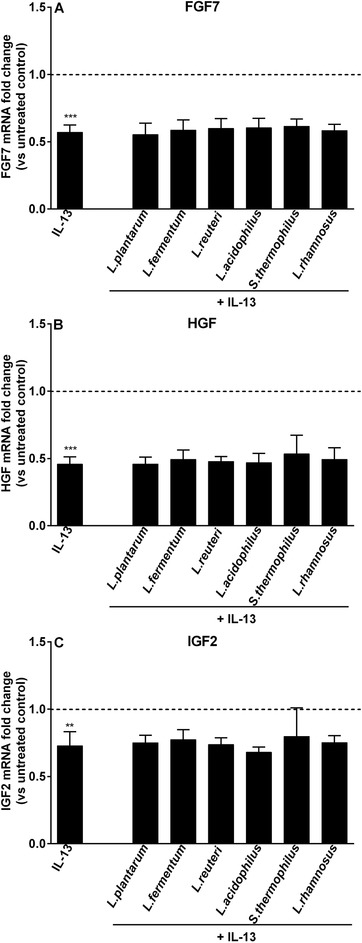
LAB‐conferred modulation of growth factor genes transcription in fibroblasts co‐cultured with goblet cells during IL‐13 challenge. CCD‐18Co fibroblasts were cultured with LS174T goblet‐cells (co‐culture), which were concomitantly stimulated with LAB (2.3 × 10^7^ CFU mL^–1^) and IL‐13 (25 ng mL^–1^) for 48 h. FGF7, HGF, and IGF2 expression levels in CCD‐18Co fibroblasts were assessed at the end of stimulation. Results are calculated as fold change in mRNA levels of growth factor genes against co‐culture untreated control group, and represent mean and SD of four independent experiments. One‐way ANOVA with Bonferroni multiple comparisons test was used to determine statistical significance (*vs co‐culture untreated control; **p < 0.01; ***p < 0.001).

In addition to TNF‐α, we also examined whether fibroblasts affect T helper 2 cytokine IL‐13‐conferred effects on goblet cell‐associated genes. It was observed that IL‐13 differently modulated goblet cell‐related genes as compared to TNF‐α. In both LS174T monocultures and LS174T/CCD‐18Co co‐cultures, expression of all five tested genes was remarkably elevated in LS174T goblet‐cells exposed to IL‐13 (**Figure** 6). As shown in Figure S1, Supporting Information, IL‐13‐induced increased expression levels of RETNLB, CHST5, and GAL3ST2 were significantly declined by the presence of fibroblasts (*p *< 0.01 for both RETNLB and GAL3ST2, *p *< 0.001 for CHST5, monoculture IL‐13 vs co‐culture IL‐13). Moreover, fibroblasts induced a trend of decreasing IL‐13‐induced upregulated TFF3 expression (*p* = 0.054, monoculture IL‐13 vs co‐culture IL‐13; Figure S1, Supporting Information). For fibroblast‐related growth factor genes (FGF7, HGF, and IGF2), their transcription was all significantly suppressed by IL‐13 stimulation in LS174T goblet‐cells as compared to co‐culture untreated control group (*p *< 0.001 for both FGF7 and HGF; *p *< 0.01 for IGF2; **Figure** 7).

Next, we studied whether LAB differently regulated the expression of goblet cell‐related genes in the absence or presence of fibroblasts during IL‐13 exposure. As shown in Figure 6, different regulatory effects of LAB on goblet cell‐related genes were achieved with or without the presence of CCD‐18Co fibroblasts during IL‐13 stimulation. For instance, IL‐13‐stimulated MUC2 expression was effectively lowered by *S. thermophilus* CCFM218 only when fibroblasts were absent (*p *< 0.05 vs monoculture IL‐13; Figure 6A). Moreover, *L. rhamnosus* CCFM237 exerted effective suppression of IL‐13‐induced elevated TFF3 expression in the presence of fibroblasts (*p *< 0.05 vs co‐culture IL‐13; Figure 6B). Similar inhibitory effects of LAB during IL‐13 exposure was observed with *L. fermentum* CCFM787 treatment on RETNLB expression (*p *< 0.05 vs co‐culture IL‐13; Figure 6C). LAB did not modify IL‐13‐induced increased expression of CHST5 and GAL3ST2 in both monoculture and co‐culture systems (Figure 6D,E). When evaluating the effect of LAB application on the expression of fibroblast‐related genes in the LS174T/CCD‐18Co co‐culture system, we found that LAB did not confer regulatory effects on growth factor genes (Figure 7).

### 3.6 LAB Could Not Effectively Dampen Tm‐Triggered Dysregulation of Goblet Cell‐Related Genes Expression in the Absence and Presence of Fibroblasts

**Figure 8:**
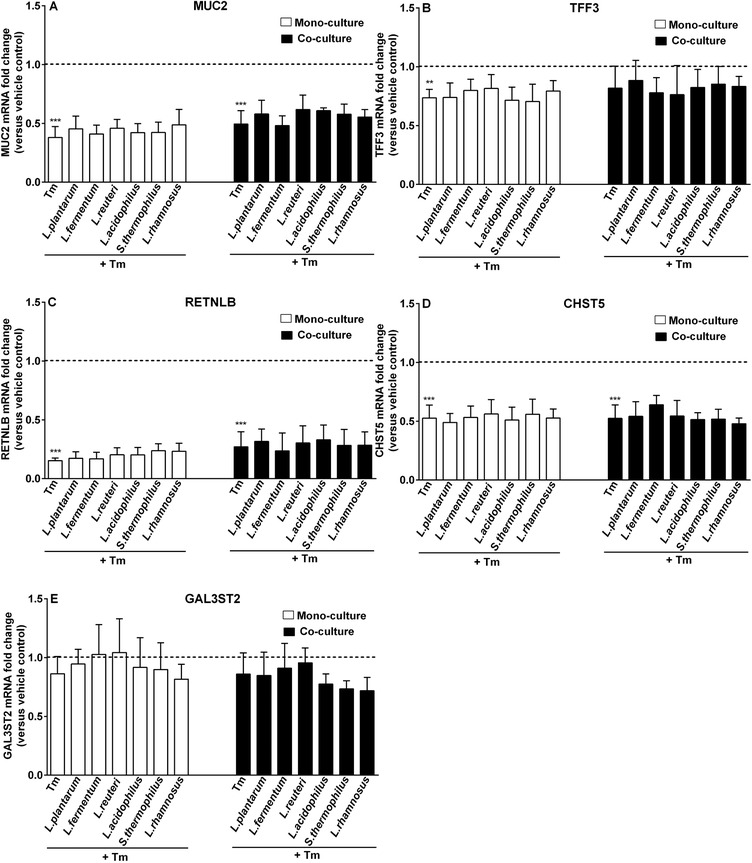
LAB did not improve Tm‐elicited defective transcription of goblet cell‐related genes in the presence and absence of fibroblasts. LS174T goblet‐cells cultured alone (monoculture) or with CCD‐18Co fibroblasts (co‐culture) were first treated with LAB strains (2.3 × 10^7^ CFU mL^–1^) for 24 h and subsequently stimulated with Tm (1 µg mL^–1^) for 12 h. Afterward, mRNA levels of MUC2, TFF3, RETNLB, CHST5, and GAL3ST2 in LS174T goblet cells were quantified and presented as fold change normalized to respective vehicle control (DMSO‐treated) groups (monoculture or co‐culture vehicle control). Results are shown as mean and SD of five independent experiments. One‐way ANOVA with Bonferroni multiple comparisons test was used for statistical comparisons (*vs vehicle control; **p < 0.01; ***p < 0.001).

**Figure 9:**
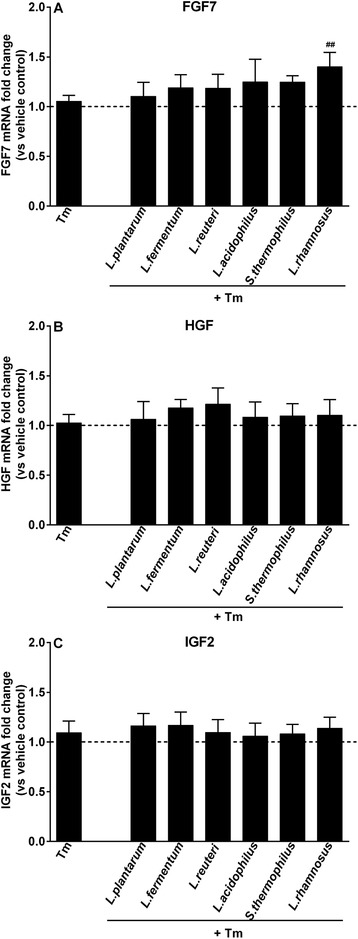
LAB‐exerted modulation of growth factor genes expression in fibroblasts during Tm challenge when in co‐culture with goblet cells. CCD‐18Co fibroblasts were cultured with LS174T goblet‐cells (co‐culture), which were first treated with LAB (2.3 × 10^7^ CFU mL^–1^) for 24 h, followed by 12 h stimulation with Tm (1 µg mL^–1^). FGF7, HGF, and IGF2 expression levels in CCD‐18Co fibroblasts were examined at the end of stimulation. Results are calculated as fold change in mRNA levels of growth factor genes against co‐culture vehicle control group, and represent mean and SD of five independent experiments. One‐way ANOVA with Bonferroni multiple comparisons test was used to determine statistical significance (^#^vs co‐culture Tm group; ^##^ *p *< 0.01).

Tm is considered to impair goblet cell functions and disrupt mucin biosynthesis via inducing ER stress.^14^ We first investigated whether fibroblasts impact Tm‐induced defects in goblet cells. As shown in **Figure** 8, TFF3 expression was significantly diminished by Tm only in LS174T monoculture system (*p *< 0.01 vs monoculture vehicle control). Tm stimulation remarkably inhibited the expression of MUC2, RETNLB, and CHST5 under both monoculture and co‐culture conditions (*p *< 0.001 vs respective vehicle controls; Figure 8A, C, and D), and Tm‐inhibited MUC2 expression were significantly upregulated in the presence of fibroblasts (*p *< 0.05, monoculture Tm vs co‐culture Tm; Figure S1, Supporting Information). Additionally, the expression of growth factor genes (FGF7, HGF, and IGF2) in CCD‐18Co fibroblasts was also measured but all three growth factor genes remained unchanged in response to Tm challenge (**Figure** 9).

Next, we explored whether LAB pretreatment prior to Tm exposure could dampen Tm‐induced goblet cell dysfunction in the presence of fibroblasts. As shown in Figure 8, LAB pretreatment did not effectively modulate Tm‐induced diminution in goblet cell‐associated genes expression irrespectively of whether fibroblasts were present or not (Figure 8A‐D). For fibroblast‐related growth factor genes, only FGF7 transcription was enhanced by *L. rhamnosus* CCFM237 (*p *< 0.01 vs co‐culture Tm; Figure 9A)

## 4 Discussion

In the present study, we confirm the presence of crosstalk between intestinal subepithelial fibroblasts and goblet cells and its impact on the expression of mucus barrier‐associated genes (MUC2, TFF3, RETNLB, CHST5, and GAL3ST2) in goblet cells. Fibroblasts not only modulated goblet cell‐related gene expression under homeostatic conditions but also during application of stressors such as cytokines and naturally occurring microbial derived mucin synthesis inhibitors such as Tm. Moreover, fibroblasts changed LAB‐induced modulation of mucus‐related gene expression in goblet cells, and their effects were highly LAB‐strain and species dependent. This emphasizes the importance of using in vitro selection tools for selecting and designing effective food‐based strategies to reinforce mucus function.

Under homeostatic conditions, fibroblasts differentially regulated the expression of different mucus barrier‐related genes and their impacts were LAB species‐dependent. In the absence of LAB stimulation, fibroblasts significantly enhanced MUC2 expression but decreased the expression of RETNLB and GAL3ST2. Inconsistency between the changing trend of MUC2 and of RETNLB expression has been observed in previous studies as well.^28^, ^29^ For MUC2, similar enhancing effects on its expression by fibroblasts were observed in the presence of specific LAB strains, i.e., *S. thermophilus* CCFM218 and *L. rhamnosus* CCFM237. However, this was different for GAL3ST2 in the presence of specific LAB strains (*L. fermentum* CCFM787, *L. reuteri* CCFM14, *L. acidophilus* CCFM137, and *S. thermophilus* CCFM218) that enhanced instead of downregulated GAL3ST2 expression following 48 h of LAB treatment when fibroblasts were present. This was a fibroblast dependent effect as it was not observed in the absence of colonic fibroblasts. *L. reuteri* CCFM14 induced enhanced GAL3ST2 expression in goblet cells that might be attributed to upregulation of HGF expression in fibroblasts by this strain as HGF has been shown to enhance the production of rat stomach mucin.^23^

Not only MUC2 and GAL3ST2 but also CHST5 expression was enhanced by specific LAB strains (*L. acidophilus* CCFM137 and *L. rhamnosus* CCFM237) in the presence of fibroblasts. This can not be explained by the regulatory growth factor genes (FGF7, HGF, and IGF2) that we studied in fibroblasts since we did not observe relevant alterations of expression of these genes in response to those CHST5‐promoting strains. Furthermore, in the absence of colonic fibroblasts, when goblet cells were cultured with the LAB strains *L. plantarum* CCFM634, *L. fermentum* CCFM787, *L. acidophilus* CCFM137, *S. thermophilus* CCFM218, *L. reuteri* CCFM14, and *L. rhamnosus* CCFM237, TFF3, and RETNLB expression and their associated pathways were specifically augmented. These pathways were blocked, however, by the fibroblast as TFF3 and RETNLB expression was not increased by any of the aforementioned LAB strains in the presence of fibroblasts. As the expression of the tested fibroblast genes following stimulation by these TFF3/RETNLB‐enhancing strains could not explain this inhibitory effects of fibroblast on TFF3 and RETNLB expression, the responsible fibroblast‐derived factors remain to be identified.

During exposure to the tested stressors TNF‐α, IL‐13, and Tm, fibroblasts impacted different stressor‐conferred effects on goblet cell‐related genes. As stressor we chose TNF‐α as it has been shown to be a cytokine contributing to the pathogenesis of inflammatory bowel disease and can dysregulate mucus synthesis.^6^, ^30^ Fibroblasts were found to further upregulate TNF‐α‐induced enhanced MUC2 expression. During TNF‐α challenge, fibroblasts also strongly impacted goblet cell‐related genes in the presence of the LAB strain *L. rhamnosus* CCFM237. This strain induced downregulation of TFF3 transcription in the absence of fibroblasts and this was not observed in the presence of fibroblasts. This cannot be ascribed to the effects of the examined fibroblast growth factor genes as the expression of these fibroblast genes remained unchanged when in co‐culture with *L. rhamnosus* CCFM237‐stimulated goblet cells. Instead we found that TNF‐α stimulation led to decreased IGF2 expression in fibroblasts, which may be due to the direct interaction of fibroblasts with TNF‐α. TNF‐α is known to cause compromised gut barrier^31^ that might also involve effects on colonic fibroblasts that subsequently impacts subepithelial cell functions.

In addition, cytokine IL‐13 was studied as it mediates host resistance against intestinal nematodes through supporting mucus production.^32^ Fibroblasts downregulated IL‐13‐induced elevated expression levels of RETNLB, CHST5, and GAL3ST2. We found that all three tested regulatory genes in fibroblast were suppressed by IL‐13. Furthermore, in the presence of LAB stimulation, fibroblasts seemed to enhance the inhibitory potentials of specific LAB strains such as of *L. rhamnosus* CCFM237 and *L. fermentum* CCFM787 on IL‐13‐induced increases in TFF3 and RETNLB expression. The inhibitory effects of these strains were only found when fibroblasts were present. However, effects of fibroblasts were strain dependent as the presence of fibroblasts attenuated rather than promoted *S. thermophilus* CCFM218‐conferred downregulation of IL‐13‐induced heightened MUC2 expression.

The mucin glycosylation inhibitor Tm induces ER stress in goblet cells and thereby leads to defective mucin synthesis and impaired mucus barrier function.^33^ Tm stimulation‐induced declined MUC2 expression levels were elevated by fibroblasts. It was also observed that Tm strongly suppressed TFF3 transcription in goblet cell monoculture system, which was not observed in the presence of fibroblasts. In the presence of LAB, fibroblasts had no further influence on Tm‐induced impaired gene expression profile in goblet cells. Of note, during Tm stimulation specific LAB strain, i.e., *L. rhamnosus* CCFM237 increased FGF7 transcription, which was not observed during cytokine (TNF‐α or IL‐13) challenge. Moreover, this promoting effect of *L. rhamnosus* CCFM237 was also not observed when Tm was not applied in goblet cells. This illustrates that regulatory properties of LAB on fibroblast genes are dependent on the applied stressor and LAB strains.

As a first start, the intestinal goblet cell monoculture model was established in a previous study as a preliminary technology platform to test the regulatory potentials of bacterial strains on goblet cell activity. Many LABs can be deleted as they have no or only minor effects in goblet cells. In order to better mimic the in vivo situation, we aimed here to develop a more complex model based on the previous monoculture model. Therefore, in the current study, we have focused on the contribution of fibroblasts on LAB‐exerted regulatory effects on goblet cell activity. Our findings demonstrate functional interactions between intestinal fibroblasts and goblet cells by impacting mucus function‐associated genes during exposure to inflammatory stressors or natural, food‐occurring mucin synthesis inhibitors. Moreover, fibroblasts were shown to impact the regulatory effects of various LAB strains on the expression of goblet cell‐related genes, and the effects of fibroblasts are contingent on the applied mucus barrier stressor and bacterial species‐specific. Our results illustrate the necessity of including fibroblasts‐conferred effects on goblet cell functions, and suggest that the co‐culture model of intestinal goblet cells and colonic fibroblasts is more suitable than the goblet cell monoculture model. To the best of our knowledge, we are the first to systematically investigate the impact of fibroblast‐goblet cell crosstalk on mucus barrier‐related genes and its role in LAB‐exerted mucus‐regulatory effects. This knowledge might contribute to the design of novel food based approaches to regulate gut barrier function.

## Conflict of Interest

The authors have declared no conflict of interest.

## Supporting information

Supporting Information
