# Supplementary material for: Fibroblasts Impact Goblet Cell Responses to Lactic Acid Bacteria After Exposure to Inflammatory Cytokines and Mucus Disruptors
Source: Mol Nutr Food Res. 2019 Apr 22;63(12):1801427. doi: 10.1002/mnfr.201801427 (PMC6618108; doi:10.1002/mnfr.201801427)
Supplement: Supplementary file 1 — Supporting Information [file MNFR-63-na-s001.docx]

**Fibroblasts impact goblet cell responses to lactic acid bacteria after exposure to inflammatory cytokines and mucus disruptors**

Chengcheng Ren^1,2^, Jelleke Dokter-Fokkens^1^, [Susana Figueroa Lozano](http://www.rug.nl/staff/f.s.figueroa.lozano/research)^1^, Qiuxiang Zhang^2^, Bart J. de Haan^1^, Hao Zhang^2^, Marijke M. Faas^1^, and Paul de Vos^1^

^1^Immunoendocrinology, Division of Medical Biology, Department of Pathology and Medical Biology, University of Groningen and University Medical Center Groningen, Hanzeplein 1, 9700 RB Groningen, The Netherlands.

^2^School of Food Science and Technology, Jiangnan University, 1800 Lihu Road, Wuxi 214122, China

**^*^Correspondence:** Chengcheng Ren (Email: [s.ren@umcg.nl](mailto:s.ren@umcg.nl))

**Supporting information**

**(Legend to Figure S1)**

**Figure S1. Expression of goblet cell-associated genes was differentially impacted by fibroblasts during exposure to cytokines or the mucin synthesis inhibitor Tm.** During cytokine (TNF-α or IL-13) challenge, LS174T goblet-cells were treated with TNF-α (50 ng/ml) for 24 h or with IL-13 (25 ng/ml) for 48 h in the absence (mono-culture) and presence of CCD-18Co fibroblasts (co-culture). For Tm challenge, LS174T goblet-cells cultured alone (mono-culture) or with CCD-18Co fibroblasts (co-culture) were treated with Tm (1 μg/ml) for 12 h. MUC2, TFF3, RETNLB, CHST5, and GAL3ST2 mRNA expression levels were measured at the end of treatment. Data were normalized to the housekeeping gene GUSB and presented as 2^-△Ct^. Results shown are mean and SD of five independent experiments. Statistical significance between the mono-culture group and the co-culture group during exposure to individual stressors was analyzed by paired t test( **^#^**= *p* < 0.05; **^##^**= *p* < 0.01; **^###^**= *p* < 0.001).
